# Relevance of lesion size in navigator-triggered and free-breathing diffusion-weighted liver MRI

**DOI:** 10.1007/s00330-024-11063-1

**Published:** 2024-09-17

**Authors:** Bianca Reithmeier, Frederik B. Laun, Tobit Führes, Michael Uder, Sebastian Bickelhaupt, Marc Saake

**Affiliations:** Institut für Radiologie, Universitätsklinikum Erlangen, Friedrich-Alexander-Universität Erlangen-Nürnberg (FAU), Erlangen, Deutschland

**Keywords:** Liver, Magnetic resonance imaging, Diffusion magnetic resonance imaging, Respiratory-gated imaging techniques, Echo-planar imaging

## Abstract

**Objectives:**

The purpose of this study was to investigate the relevance of focal liver lesions (FLL) size for lesion detection comparing navigator triggering (TRIG) to free breathing (FB) liver Diffusion-weighted magnetic resonance imaging (DWI).

**Materials and method:**

Patients with known or suspected FLL were prospectively (registry number 276_19 B) included from October to December 2019 in this study, out of which 32 had liver lesions. Echo planar spin-echo DWI data both with TRIG and FB were with approximately constant acquisition times acquired at 1.5 T. Lesions were segmented in the *b* = 800 s/mm² images in both the TRIG and FB images. The lesion size, location (liver segment), liver lesion visibility, as well as contrast-to-noise ratio (CNR) were recorded. The CNR was assessed with the Wilcoxon–Mann–Whitney test and the number of visible lesions with the Fisher test.

**Results:**

Data from 43 patients (22 female) were analyzed. The mean patient age was 58 ± 14 years. A total of 885 FLL (*N*_total_) were segmented. Among these, 811 lesions (*N*_both_) were detected with TRIG and FB, 65 lesions exclusively with TRIG (*N*_TRIG_Only_), and nine exclusively in FB (*N*_FB_Only_). The largest additional lesion in TRIG/FB had a diameter of 10.4 mm/7.6 mm. The number of additional lesions detected with TRIG decreased with size. Among all lesions ≤ 4.7 mm, the relative number of additional lesions was 15.6%. Additional lesions were found in all liver segments with TRIG. In the left liver lobe, the relative proportion was 9.2%, and in the right liver lobe 5.4%. CNR and visibility were significantly higher in TRIG than in FB (*p* < 0.001). In relation to size, the difference is significant in terms of visibility and CNR for lesion diameters ≤ 8 mm.

**Conclusion:**

Respiration triggering can improve the detection of small liver lesions with diameters up to approx. 1 cm in the whole liver.

**Key Points:**

***Question*** *Can respiration triggering (TRIG) improve the detection of small FLL compared to FB diffusion-weighted imaging*?

***Findings*** *Among 885 segmented FLL, TRIG was superior to FB for lesions smaller than 8* *mm and had improved CNR and visibility*.

***Clinical relevance*** *Diffusion-weighted magnetic resonance imaging is used for the detection of focal liver lesions and image quality is influenced by breathing motion. Navigator triggering becomes more important for smaller lesions, and seems recommendable for the detection of small focal liver lesions*.

## Introduction

Diffusion-weighted (DWI) magnetic resonance imaging (MRI) is often used in imaging of the liver [1, 2]. In patients with tumor disease, it is often essential to determine to what extent the liver is affected. In particular, the prognosis of many malignant diseases depends on the extent of the affected liver parenchyma [3].

Liver DWI is affected by breathing and cardiac motion [4]. Cardiac motion particularly affects the left liver lobe, being near the heart, leading to signal dropouts [5–8]. DWI acquisitions are usually performed with a relatively large number of image averages. Thus, untriggered free breathing (FB) acquisition may lead to blurring of the lesions or copies of the lesions at adjacent slice positions. Different techniques can reduce these artifacts: the use of algorithms for motion correction [9], and breath holding (BH) [10–12]. This addresses the motion problem, but fewer images are acquired in the same scan time and BH requires the cooperation of the patient. If the breath is held for too short a time or inconsistently, the position of the liver may vary strongly, resulting in image artifacts. Patient cooperation cannot always be guaranteed in clinical practice, especially if severely diseased patients are examined. As BH seems to be less suitable than the other techniques (see discussion section of [13] and [14–16]), the question remains whether FB acquisition or navigator-triggered (TRIG) acquisition is more suitable for liver DWI of focal liver lesions (FLL). In TRIG [14, 15, 17], the acquisition time of the images is adapted to respiration, to minimize the shift of internal organs. Two factors compete here; FB allows the acquisition of more data in the same amount of time, therefore generally increasing the signal-to-noise ratio and the contrast-to-noise ratio (CNR). In contrast, TRIG extends the acquisition time—sometimes in a manner that is difficult to predict—but mostly solves breathing motion-related problems. Which of the two factors is of higher importance is not completely evident a priori.

Choi et al found little difference between the lesion detection and characterization ability of the two approaches, with an increased time-efficiency of the FB acquisition [16]. Takayama et al reported no significant differences between FB and TRIG concerning the lesions CNR [18]. Szklaruk et al found better image quality with TRIG, but they did not evaluate the lesion conspicuity [19]. Nasu et al indicated a better lesion contrast with external breath triggers than with FB and noted more misregistrations with FB acquisitions [20].

The discrepancies between these studies might be explained at least partly by different lesion sizes (Nasu et al: range 0.8–4.0 cm; mean 1.5 cm. Choi et al: range 1.3–6.0 cm; mean 2.7 cm). The ongoing increase in image quality due to technical advances allows the assessment of smaller and smaller lesions. Hence the question about the relevance of FB or TRIG acquisition for smaller lesions detection increases. Naturally, one would assume that TRIG is more relevant for smaller lesions.

A prior study has recently shown that more small FLLs could be detected with TRIG acquisition [13]. However, lesions were only classified in two categories (smaller or larger than 1 cm). Moreover, due to the large number of lesions, only the difference in visible lesions was counted, but not the absolute numbers.

The aim of this work was to perform a detailed study on the relevance of lesion size in liver DWI with respect to fractions of missed lesions and the CNR. The underlying hypothesis is that TRIG and FB techniques perform differently.

## Materials and methods

### Study population

This study has been approved by the local ethics committee and written informed consent was obtained in all patients. Patients aged at least 18 years and with known or suspected liver lesions had been prospectively (registry number 276_19 B) included from October to December 2019. Further inclusion criteria had been the tolerability of the longer examination by the study sequence. Exclusion criteria had been those against a conventional MRI examination—ferromagnetic implants, large tattoos, and claustrophobia. The additional acquisitions for the study had been performed during the regular MRI appointment in the clinical routine. The same source data (all 43 patients) had been used in a previous evaluation which did not investigate the explicit sizes and positions of the lesions [13].

### MRI

All measurements were performed with a clinical 1.5-T scanner (MAGNETOM Aera, XQ gradient with max. gradient strength 45 mT/m and max. slew rate 200 T/m/s, Siemens Healthcare) using a vendor-provided 18-channel anterior body coil in combination with a vendor-provided 32-channel spine array coil. A single refocused echo planar imaging (EPI) product sequence was used for DWI. The acquisition parameters are summarized in Table 1. We kept the repetition time fixed for both sequences to ensure the use of four concatenations for both the FB and the TRIG acquisition.

**Table 1:** MRI sequence parameters for TRIG and FB DWI sequences

Sequence	DWI EPI
Repetition time (ms)	1800
Echo time (ms)	56
Pixel size (mm²)	1.5 × 1.5 interpolated from 3 × 3
Slice thickness (mm)	5
Field of view (read × phase; mm²)	380 × 309
Phase direction	Anterior, posterior
Phase resolution	100%
Partial Fourier	Off
Slice distance	20%
Number of slices	39 (axial)
Parallel imaging	GRAPPA × 2, 36 reference lines
Bandwidth (Hz/pixel)	2298
Echo spacing (ms)	0.5
*b*-values (s/mm²)	50, 800
Averages (b50, b800)	FB: 2, 7 TRIG: 1, 2
Diffusion mode	3-scan trace
Diffusion scheme	Single-refocused (“monopolar”), with “dynamic field correction“ to compensate for eddy current-induced image distortions
Nominal acquisition time (min:s)	3:51 (FB), 2:30 (nominal, TRIG)
Mean of acquisition time estimated with DICOM tag “acquisition time” (min:s)	3:27 (FB), 4:09 (TRIG)
Trigger for TRIG	TRIG: PACE, scout TR = 150 ms, acquisition window = 35%, liver dome scout
Concatenations	4
Surface coil intensity correction	“Prescan normalize”
Fat saturation	SPAIR and gradient reversal

*FB* free-breathing acquisition, *TRIG* navigator-triggered acquisition

The measurements were performed once with FB acquisition and once with TRIG. For TRIG, the prospective acquisition correction (PACE) technique provided by the vendor was used. The nominal acquisition time was adapted for TRIG to keep the actual acquisition time roughly equal to the FB sequence. As the real acquisition times can depend on the breathing pattern, they were determined after the exams by determining the smallest and the largest value of the DICOM tag “(0008, 0032) acquisition time” of all images of a series. This tag describes when the data acquisition started and is therewith a good proxy for the assessment of the total acquisition time for EPI images, but it neglects the time needed for preparatory scans. The preparatory scans, i.e., the acquisition of the GRAPPA reference data and the dummy scans, took approximately 30 s.

### Image analysis

The datasets were checked for sufficient image quality.

FLL were segmented separately in FB and TRIG images using the Medical Imaging Interaction Toolkit (MITK, v2021.02) using the images (not the ADC maps) acquired at *b* = 800 s/mm^2^ by a final grade medical student (B.R.), guided and assisted by a board-certified radiologist (M.S. with over 15 years of experience in abdominal imaging) and a physicist (T.F., 4 years experience in liver DWI). Likely benign FLLs such as cysts or hemangiomas were excluded based on the judgment of board-certified radiologists made in the clinical routine in clinical reports. Additionally, these were identified using all available clinical image data including T2w and contrast-enhanced T1w images. In the segmentation process, it was allowed to view the full clinical MRI dataset for lesion characterization.

Lesions that were only visible in one image set were rated as additional lesions. Here, adjacent slices were inspected in both DWI techniques to rule out missing a lesion due to breathing-induced shifts in slice position. An identical image windowing was set and kept for both acquisition techniques for each patient, thus aiming at a patient-specific optimal visualization. During segmentation, it was made sure that the boundaries of the lesion were excluded to minimize partial volume effects. A lesion was always completely segmented. That is, central necroses were also included. If there were overlaps with vessels, these were not segmented. After segmenting all lesions, a quality check was performed to make sure that lesions that had been marked as additional lesions were indeed only visible in one dataset (FB or TRIG). For this, the images were inspected a second time (with potentially changed windowing, if deemed necessary) by M.S. and potential errors were corrected. In case of doubt, hardly visible lesions were evaluated as detectable and not as additional lesions.

For each lesion, the corresponding liver segment was also noted. If a lesion covered more than one liver segment, the segment that contained the largest proportion of the lesion was chosen for the evaluation.

The lesions were categorized into size ranges according to the respective number of voxels (voxels: 1–5, 6–10, 11–25, 26–50, 51–75, 76–100, 101–250, 251–500, > 500). This size was converted into a lesion diameter by assuming a spherical shape. To describe the lesions three-dimensionally (i.e., to take all layers into account), the diameter was not simply specified as a manual axial measurement. The size of lesions segmented in both images was assigned based on the sizes of the TRIG images. In order to make a fair comparison, the number of additional lesions was defined by *N*_∆_ = *N*_TRIG_Only_ − *N*_FB_Only_, which is the difference between the number of lesions only seen in TRIG and the number of lesions only seen in FB data.

For the calculation of CNR, the standard deviation of noise can be used. However, in multi-channel MRI, the standard deviation of noise cannot be simply evaluated, as it generally depends on the image position. Therefore, following [13, 14, 16, 21, 22], the standard deviation ${\sigma }_{{{{\rm{ref}}}}}$ of the liver parenchyma was used for CNR calculation by placing a 10 cm² reference region of interest (ROI) in both images. This ROI was drawn on a representative slice, preferably in the right lobe of the liver if available, sparing major vessels and lesions.

The CNR was then calculated by the difference of the average signal in the 3D lesion segmentation (${{S}}_{0}$) and the mean value of the reference ROI (${{S}}_{{\mbox{ref}}}$) divided by the signal’s standard deviation in the reference ROI (*σ*_ref_).

$${{{\rm{CNR}}}}=\frac{{S}_{0}-{S}_{{{{\rm{ref}}}}}}{{{\sigma }}_{{{{\rm{ref}}}}}}$$

CNR was calculated for each lesion. Negative CNRs sometimes occurred due to numerical errors for small and faintly visible lesions, but were not excluded from the analyses. The CNR of FLLs invisible with one acquisition technique was set to zero for this technique.

### Statistical analysis

Statistical analysis was performed with MATLAB Release 2020b (MathWorks, Inc.) by B.R. and T.F. The Shapiro–Wilk test was used to test the CNR values for normal distribution. Because this test did not result in a normal distribution, the Wilcoxon–Mann–Whitney test was conducted to assess significant differences between the CNR of FB and TRIG datasets. The significance of the number of lesions was tested using the Fisher test. This was done by comparing the visibility to FB and TRIG. A *p*-value < 0.05 was considered significant.

## Results

### Patients

**Fig. 1:**
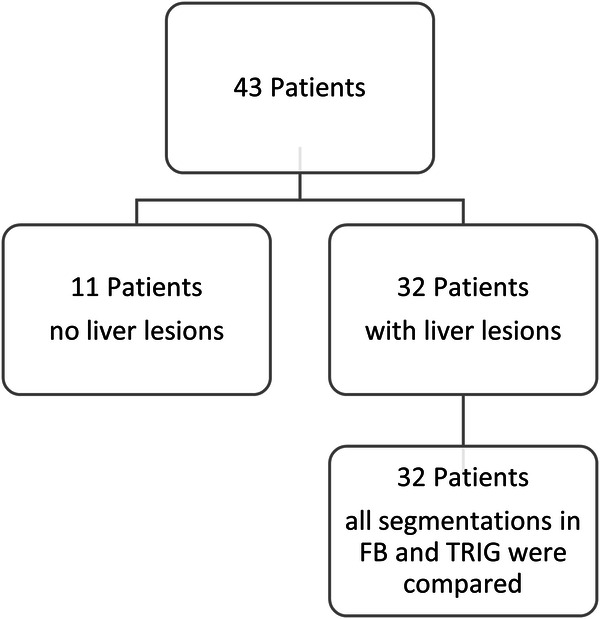
Study flow chart. FB, free-breathing acquisition; TRIG, navigator-triggered acquisition

In the here presented evaluation, data from 43 patients were analyzed. The average age of the patients was 58 years (range 24–81 years). All liver MRI scans had been successfully and completely performed. Image data quality was deemed adequate in all patients and thus all patients were included in the study. Of the 43 patients, 11 patients had no liver lesions, thus FLLs could be compared in 32 patients. Figure 1 and Table 2 show the distribution of the study population. The patients had the following diseases: carcinoma of the breast (2), colorectal carcinoma (14), epithelioid sarcoma (1), esophageal carcinoma (1), leiomyosarcoma (1), neuroendocrine tumor (19), pancreatic carcinoma (1), thyroid cancer (1), uveal melanoma (2), and lung cancer (1).

**Table 2:** Baseline demographics of participants

Age (y)	58.3 ± 14 years
Sex (M/F)	
Male	21
Female	22
Hemihepatectomy	
Left	1
Right	5
Diseases	
Carcinoma of the breast	2
Colorectal carcinoma	14
Leiomyosarcoma	1
Esophageal carcinoma	1
Epithelioid sarcoma	1
Pancreatic carcinoma	1
Thyroid cancer	1
Neuroendocrine tumor	19
Uveal melanoma	2
Lung cancer	1

### Representative images

**Fig. 2:**
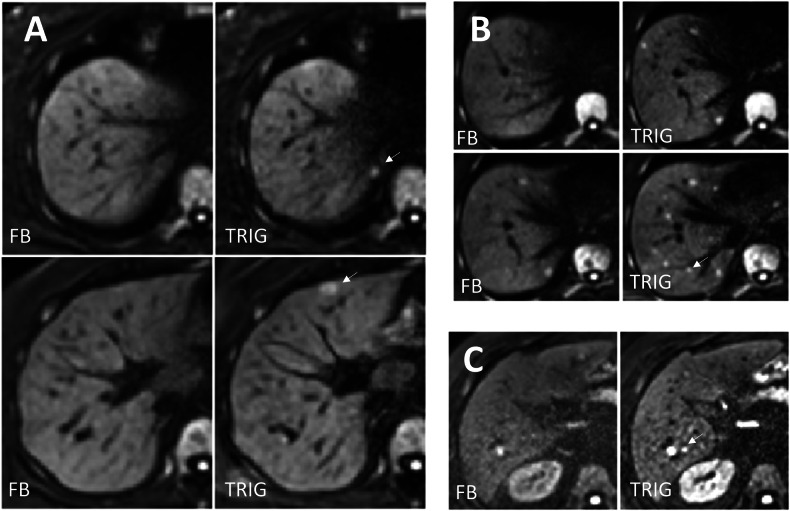
Liver DWI b800 images (**A**) of a 76-year-old patient with metastatic colorectal carcinoma, (**B**) of a 47-year-old patient with metastatic thyroid carcinoma, and (**C**) of a 60-year-old patient with metastatic neuroendocrine tumor. Different slices are shown for FB (left) and TRIG (right). The lesion that is only visible in TRIG images is marked with an arrow. All other lesions are recognizable with both techniques on neighboring layers, which are not shown. FB, free-breathing acquisition; TRIG, navigator-triggered acquisition

**Fig. 3:**
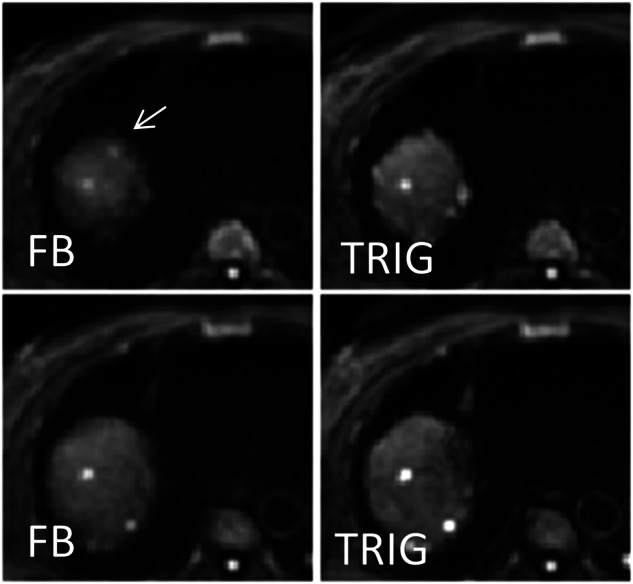
Liver DWI b800 images of a 79-year-old patient with metastatic neuroendocrine tumor. Two adjacent slices are shown for FB (left) and TRIG (right). The lesion is only visible in FB and is marked with a thin arrow. Other lesions are recognizable in neighboring layers. FB, free-breathing acquisition; TRIG, navigator-triggered acquisition

Representative patient images are shown in Fig. 2 and Supplemental Fig. 1. The arrows indicate the lesions only visible in TRIG but not in FB data. Figure 3 shows an example of a lesion marked with a thin arrow that can only be seen in the FB images. The pictures give a representative impression of TRIG and FB image quality. The TRIG images in these examples appear sharper while the FB images appear less noisy.

### Quantitative analysis

**Fig. 4:**
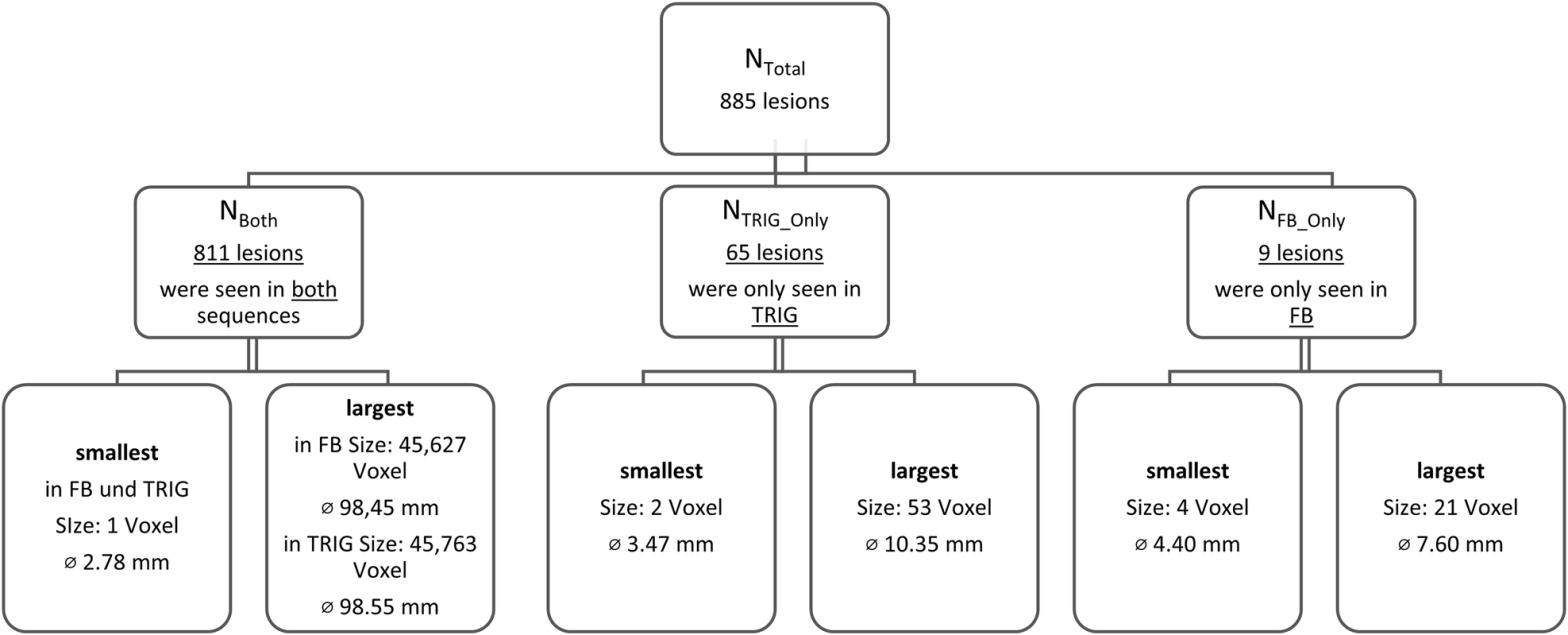
Overview of number of lesions in TRIG and FB data. Additionally, the largest and smallest sizes (total of voxels) and the respective diameter in millimeters are given. For the calculation of the diameter, the lesion was assumed to be spherical. FB, free-breathing acquisition; TRIG, navigator-triggered acquisition

A total of 885 lesions (*N*_Total_) were detected, 65 of these were exclusively visible in TRIG (*N*_TRIG_Only_), and nine exclusively in FB (*N*_FB_Only_). 811 lesions (*N*_Both_) were segmented in both images. This results in 1696 (2*N*_Both_ + *N*_FB_Only_ + *N*_TRIG_Only_) segmentations. The largest segmented lesion had a diameter of approximately 98.5 mm in TRIG and FB images. The largest lesion exclusively seen in TRIG data had a diameter of 10.4 mm. The largest lesion exclusively seen in FB data had a diameter of 7.6 mm. Figure 4 illustrates the data and gives a detailed overview of the number and size of the segmentations. In the segmentation process, it was noticed that the size of the segmentations sometimes differed greatly in the two imaging techniques. This is because the lesions were often seen in a different number of slices. Frequently, lesions were only visible in one slice.

Table 3 shows the number of segmentations per size range. Additionally, the percentage of lesions that were seen only in TRIG data or in FB data is stated. The percentage was highest for the smallest lesion size and monotonically decreased with lesion size. It was roughly by a factor of 6–10 higher for lesions only seen in TRIG data compared to FB data.

**Table 3:** Number of lesions in different size ranges: the number of lesions (*N*_both_ lesions that can be seen in TRIG and FB data, *N*_TRIG_Only_ lesions that were only seen in TRIG data, *N*_FB_Only_ lesions that were only seen in FB data, *N*_∆_ = *N*_TRIG_Only_ −  *N*_FB_Only_, as well as the relative part in percent

Size in voxel	⌀ in mm	*N*_Both_	*N*_TRIG_Only_	$\frac{{{{\boldsymbol{N}}}}_{{{{\mathbf{TRIG}}}}\_{{{\mathbf{Only}}}}}}{{{{\boldsymbol{N}}}}_{{{\mathbf{both}}}}}$	*N*_FB_Only_	$\frac{{{{\boldsymbol{N}}}}_{{{{\mathbf{FB}}}}\_{{{\mathbf{Only}}}}}}{{{{\boldsymbol{N}}}}_{{{\mathbf{both}}}}}$	${{{{\boldsymbol{N}}}}_{{{\mathbf{\Delta }}}}}$ = *N*_TRIG_Only_ − *N*_FB_Only_	$\frac{{{{\boldsymbol{N}}}}_{{{\mathbf{\Delta }}}}}{{{{\boldsymbol{N}}}}_{{{\mathbf{both}}}}}$
1–5	0–4.7	225	41	18.2%	6	2.7%	35	15.6%
6–10	4.8–5.9	176	14	8.0%	2	1.1%	12	6.8%
11–25	6.0–8.0	209	8	3.8%	1	0.5%	7	3.3%
26–50	8.1–10.1	79	1	1.3%	0	0%	1	1.3%
51–75	10.2–11.6	26	1	3.9%	0	0%	1	3.8%
76–100	11.7–12.8	19	0	0%	0	0%	0	0%
101–250	12.9–17.4	40	0	0%	0	0%	0	0%
251–500	17.5–21.9	18	0	0%	0	0%	0	0%
501–50,000	22.0–40	19	0	0%	0	0%	0	0%

For the conversion from size in voxel to diameter in mm, a spherical shape was assumed

*FB* free-breathing acquisition, *TRIG* navigator-triggered acquisition

**Fig. 5:**
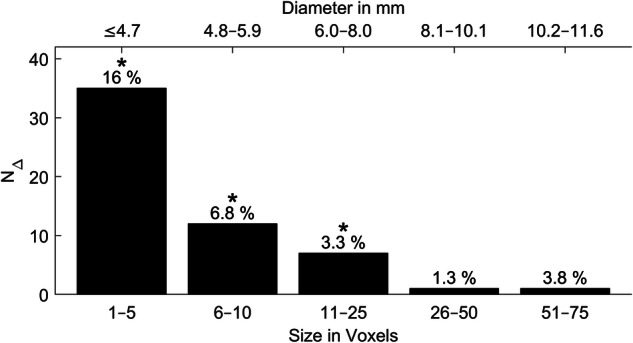
Absolute number of additional lesions (*N*_∆_ = *N*_TRIG_Only_ − *N*_FB_Only_) over the size in voxels and the diameter in mm. The relative parts of additional lesions related to all segmentations $(\frac{{{N}}_{\triangle }}{{{N}}_{{{\rm{both}}}}})$ in the relevant size range are stated as percentage numbers. The stars indicate significant differences between the visibility of TRIG and FB. FB, free-breathing acquisition; TRIG, navigator-triggered acquisition

Figure 5 shows the absolute number of additional lesions *N*_∆_ = *N*_TRIG_Only_ − *N*_FB_Only_ vs. the size in voxels and the diameter in mm. Above each bar in the diagram, the relative proportion of *N*_∆_ in relation to all lesions segmented in this size range is indicated. There were many small additional lesions; on the other hand, for larger lesions, fewer additional lesions were found. Particularly many additional lesions arose for diameters ≤ 8 mm. For lesion diameters ≤ 4.7 mm, 16% of lesions were additional lesions. The stars indicate significant differences between the visibility of TRIG and FB. The differences were significant for the voxel size range 1–25 (≤ 8.0 mm). Overall, the visibility of TRIG and FB was significantly higher in TRIG than in FB (*p* < 0.001).

Dichotomizing between the liver lobes, in the left liver lobe, 327 lesions were segmented in TRIG and FB images, 34 only in TRIG, and four only in FB images. This results in a relative number of 9.2% of additional lesions in the left liver lobe. In the right liver lobe, 484 lesions were segmented in TRIG and FB images. Five lesions were only visible in FB data and 31 only in TRIG data. This results in a relative number of 5.4% of additional lesions in the right liver lobe. A detailed overview of the number of lesions per liver segment is shown in Table 4.

**Table 4:** Number of lesions per liver segment

Liver segment	*N*_both_	*N*_FB_Only_	*N*_TRIG_Only_
I	15	0	1
II	95	2	11
III	52	1	5
IVa	120	0	14
IVb	45	1	3
V	121	2	7
VI	102	0	6
VII	101	1	10
VIII	160	2	8

*N*_both_: lesions seen in TRIG and FB data, *N*_TRIG_Only_: lesions seen in TRIG data, *N*_FB_Only_: lesions seen in FB data, *N*_∆_ = *N*_TRIG_Only_ − *N*_FB_Only_

*FB* free-breathing acquisition, *TRIG* navigator-triggered acquisition

Supplemental Fig. 2 shows the number of additional lesions (*N*_∆_) per liver segment. Additional lesions were found in each liver segment. In relative numbers, up to 12% of additional lesions were detected.

**Fig. 6:**
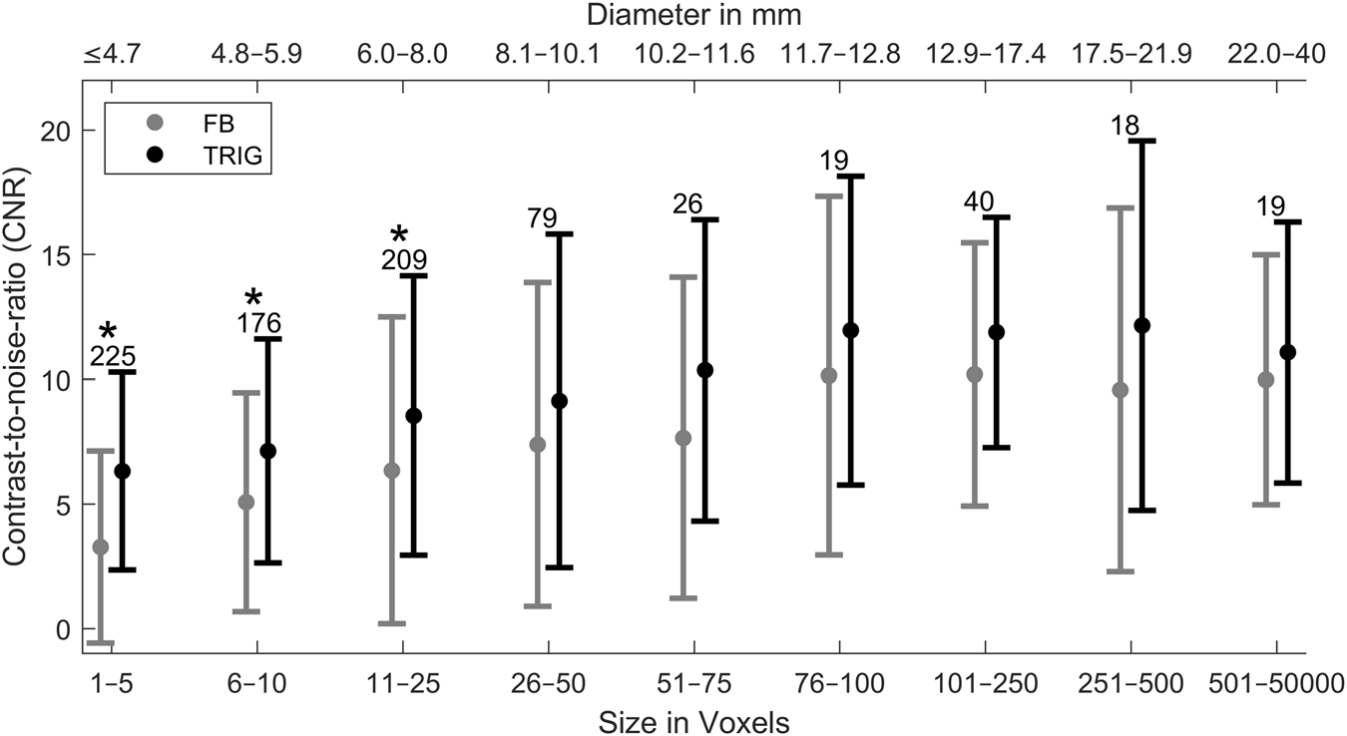
CNR as a function of size range (mean and standard deviation). The absolute number of lesions is indicated above the bars. The stars indicate significant differences between TRIG and FB. FB, free-breathing acquisition; TRIG, navigator-triggered acquisition

The CNR for the different lesion size ranges is displayed in Fig. 6. Dots indicate the mean value, and the error bars denote the standard deviations. The absolute number of lesions included in the relevant size range is stated as a numerical value above the error bars. The stars indicate significant differences between TRIG and FB. The differences are significant for the voxel size range 1–25 (≤ 8.0 mm). Overall, CNR is significantly higher in TRIG than in FB (*p* < 0.001). The mean CNR is higher for the TRIG data in all cases. The relative difference in CNR is largest for small lesions. The CNR moreover increases with lesion size.

## Discussion

In the current study, diffusion-weighted MR images acquired in free-breathing acquisition (FB) and with navigator-triggered acquisition (TRIG) were compared with a focus on the detection of small FLL. TRIG was found to increase the detection rate of small lesions. In relation to size, the difference was significant in terms of CNR for lesions ≤ 8 mm. Lesions in the left liver lobe were more prone to being missed, presumably due to the proximity to the heart and thus arising additional signal voids due to pulsation artifacts.

Thus, the question of whether the increased number of averages obtained with FB acquisition outperforms the better spatial fidelity of TRIG acquisition is decided for small lesions (at least for our setup). It does not. This is in line with the results of Szklaruk et al [19], as well as the previous evaluation, in which we already demonstrated a better delineation of the liver edge and vessel margins from the surrounding tissue using TRIG [13].

Bouchaibi et al found comparable image quality for the two acquisition settings, but they also highlighted the importance of considering lesion sizes and evaluated the detection rates for low *b*-values (10 s/mm²) [23]. We are not aware of a previous work that stratified the detection rate and the CNR of FLLs with respect to lesion size when comparing FB to TRIG acquisition for high *b*-values (like 800 s/mm²). Our findings are nonetheless in line with several previous works. On the one hand, several studies compared BH acquisitions with triggered acquisitions, which generally showed an increased performance of triggered acquisitions. For example, Kandpal et al considered the largest FLL in each patient and compared external TRIG with BH acquisition. They found that triggering leads to a significantly higher CNR than FB acquisitions [14]. Taouli et al also noted the improved image quality and increased contrast between liver and lesion with TRIG compared to BH [15]. Furthermore, there are reports on comparisons of triggering approaches and FB (as in our study). Nasu et al showed that the contrast between tumors and surrounding liver parenchyma is higher when triggering than with FB [20]. Choi et al found that FB and TRIG acquisitions are equivalent for the detection of liver lesions for lesion sizes larger than 1 cm, but FB is more time-efficient [16]. This is in line with our findings for larger lesions. They also found an increased, but not significant, CNR of FLLs with TRIG; and the increase was similar in size as we observed it for larger lesions [16]. They did not consider smaller lesions though. Another technique to deal with the breath motion is TRacking Only Navigator echo (TRON) [24, 25], where a navigator echo is used for continuous slice tracking and position correction in real-time, without using a gating window. This does not increase the scan time but results in fewer blurred images. Ivancevic et al found that images are sharper with TRON than in FB. However, triggered breathing was reported to be superior to TRON and FB acquisition in terms of image sharpness, which is consistent with our results [24].

Takayama et al also noted a slight increase in CNR of liver tumors with triggered acquisitions, but reported better scores for sharpness of the liver contour with FB acquisition [18]. The latter seems to be somewhat contradicting to other reports (e.g., [13, 19]).

The acquisition time plays an important role in clinical MRI. In the MRI sequence definition phase for this study, our focus lies on a comparable scanning time of both sequences. We reached this goal in our test population, which consisted of healthy volunteers. In the patient cohort, the mean estimated acquisition time of the TRIG sequence was almost 40 s longer compared to the FB sequence. We assume this might result from a different breathing pattern in the older patient cohort. This prolonged acquisition time is a disadvantage of the TRIG sequence. A further approach to minimize motion artifacts that might be beneficial for the assessment of small lesions is self-gating with non-cartesian k-space trajectories, e.g., as proposed in [26].

Our finding that 9.2% of lesions were missed in the left liver lobe while only 5.4% were missed in the right liver lobe can be explained reasonably by the more prominent cardiac-related signal voids in the left liver lobe. If the lesion has a decreased CNR with BH acquisition, then it is more prone to being missed if a second mechanism, like the cardiac pulsation artifact, also deteriorates its visibility and is added to the picture. The signal voids in the left liver lobe can be addressed with several techniques. One approach is ECG triggering [27, 28], which may improve quality, but the integration into the clinical routine is cumbersome and time-consuming. In addition, triggering might be impeded by fast-switching diffusion gradients, which deteriorate the signal quality of the ECG. Another possibility is flow-compensated (FloCo) diffusion encoding, which can reduce pulsation artifacts [29–32]. Alternatively, post-processing approaches have been proposed to mitigate the severeness of the signal voids by identifying (or reducing the weighting of) uncorrupted images among the repeatedly acquired diffusion-weighted images [22, 33].

Our study has some limitations. We used only one scanner from one vendor (1.5-T) at one site, which may limit the generalizability of the obtained results. For example, the navigator triggers might be implemented differently by other vendors. In addition, the malignancy of the lesions was not confirmed histologically for all lesions. A solid ground truth was thus missing. Many lesions in comparatively few patients with different tumor entities were analyzed in this study. Thus, the patients with many lesions have a very high statistical weight in the analysis. The study does not offer an assessment of the inter-observer or inter-scanner reproducibility. The manual segmentation approach might have been a source of a potential bias. Contrast-enhanced MRI might have been an option, but its sensitivity was reported to hardly outperform DWI [34]. A blinded segmentation was not possible because the two acquisitions differed so much from each other that they were distinguishable easily. For the calculation of CNR, the standard deviation of the liver parenchyma was used instead of the actual image noise. For this reason, CNR values must be regarded as an approximation to the actual values only. Nonetheless, this approach has also merit, for it potentially reflects the visibility better. Eventually, the lesion must stand out from the liver tissue and its signal fluctuations to be detectable. In general, CNR will vary depending on the used coils, sequences, and magnetic field strength. The results may well change if a field strength of 3-T, different *b*-values, or a different spatial resolution are used. The use of liver-specific contrast media may be beneficial in certain cases, but it is very limited at our institute and only used for specific questions. For this reason, we did not use it in our current study. Another limitation might be that the evaluation was performed by a senior medical student, supervised by a board-certified radiologist, which might have influenced the reported detection rates.

In summary, additional FLLs were found with respiration-triggered liver MRI acquisition throughout the entire liver. Lesions of 4.8–8 mm size were missed occasionally with the free-breathing acquisition (roughly 10%). Lesions smaller than 4.8 mm were missed regularly (almost 20%) and had a much-reduced CNR. Above 8 mm, TRIG (TRIG) and free-breathing (FB) were equally effective in detecting metastases. It thus seems not recommendable to assess small FLLs with FB DWI acquisitions.

## Supplementary information

ELECTRONIC SUPPLEMENTARY MATERIAL

## Abbreviations

BH: Breath holding
CNR: Contrast-to-noise ratio
DWI: Diffusion-weighted imaging
FB: Free-breathing acquisition
FLL: Focal liver lesions
MRI: Magnetic resonance imaging
ROI: Region of interest
TRIG: Navigator-triggered acquisition
