# Supplementary material for: Relevance of lesion size in navigator-triggered and free-breathing diffusion-weighted liver MRI
Source: Eur Radiol. 2024 Sep 17;35(4):2106–15. doi: 10.1007/s00330-024-11063-1 (PMC11913969; doi:10.1007/s00330-024-11063-1)
Supplement: Supplementary file 1 — ELECTRONIC SUPPLEMENTARY MATERIAL [file 330_2024_11063_MOESM1_ESM.pdf]

Relevance of lesion size in navigator-triggered and free-breathing diffusion-weighted liver MRI

ELECTRONIC SUPPLEMENTARY MATERIAL

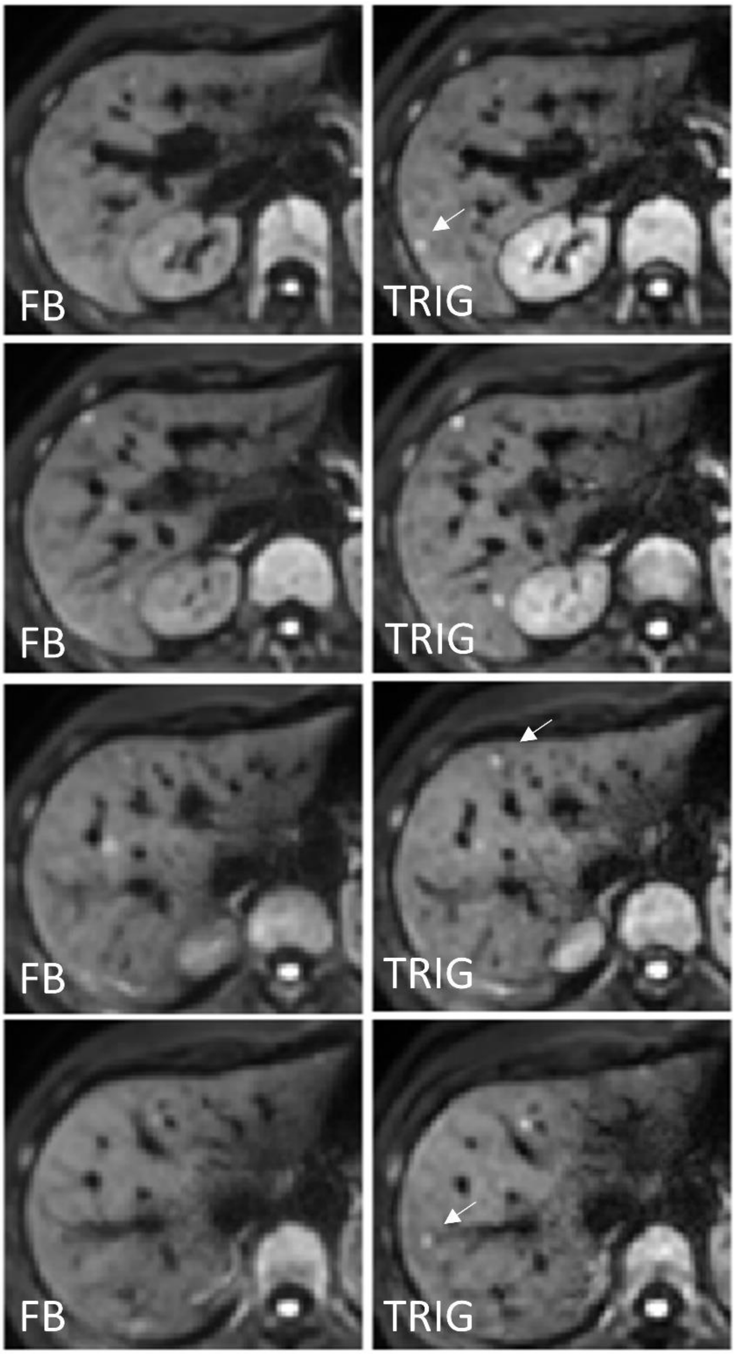

Supplemental Figure 1. Liver DWI b800 images of a 53-year-old patient with metastatic neuroendocrine tumor. Four adjacent slices are shown for FB (left) and TRIG (right). Lesions that are only visible in TRIG are marked with an arrow. Other lesions are recognizable in neighboring layers. (FB free-breathing acquisition, TRIG navigator-triggered acquisition)

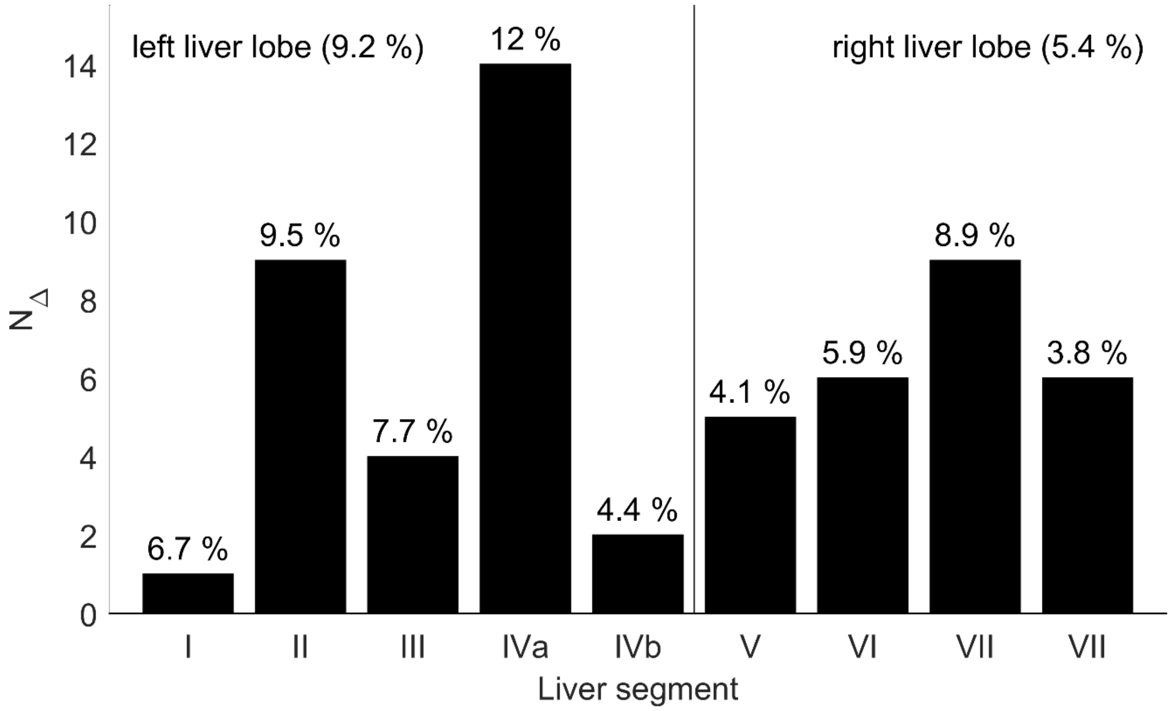

Supplemental Figure 2. Number of additional lesions (N_Δ) per liver segment. The relative percentage of additional lesions is indicated above the bars. The relative percentage for the entire left lobe is shown in the top left-hand corner, with the relative proportion of the entire right lobe shown in the top right-hand corner.
